# Experimental Galactose-1-Phosphate Uridylyltransferase (GALT) mRNA Therapy Improves Motor-Related Phenotypes in a Mouse Model of Classic Galactosemia—A Pilot Study

**DOI:** 10.3390/biomedicines13122848

**Published:** 2025-11-21

**Authors:** Olivia Bellagamba, Aaron J. Guo, Xinhua Yan, Joe Sarkis, Bijina Balakrishnan, Kent Lai

**Affiliations:** 1 Division of Medical Genetics, Department of Pediatrics, University of Utah School of Medicine, Salt Lake City, UT 84108, USA; olivia.bellagamba@hsc.utah.edu (O.B.); aaron.guo@hsc.utah.edu (A.J.G.); 2 Moderna, Inc., 325 Binney St., Cambridge, MA 02124, USA; xinhua.yan@modernatx.com (X.Y.); joe@clathrin.hms.harvard.edu (J.S.)

**Keywords:** classic galactosemia, mRNA therapy, *GALT* mRNA, motor impairment, composite phenotype scoring test, rotarod testing

## Abstract

**Background:** Despite life-saving newborn screening programs and a life-long galactose-restricted diet, many patients with classic galactosemia continue to develop long-term debilitating neurological deficits, speech dyspraxia, and primary ovarian insufficiency (POI). In an earlier study, we showed that administration of an experimental human *GALT* mRNA predominantly expressed in the liver of the *GalT* gene-trapped mouse model augmented the expression of hepatic GALT activity, which reduced build-up of galactose and its toxic metabolites not only in the liver but also in the peripheral tissues. Moreover, we showed that the administration of *GALT* mRNA in the mutant mice restored whole-body galactose oxidation (WBGO), which is a functional biomarker. **Methods:** In this pilot study, we extended our proof-of-concept efficacy studies to a disease-relevant phenotype: motor impairment. *GalT*-KO mice aged 3 and 6 weeks old administered biweekly intravenous injections of 100 µL *GALT* mRNA at a dose of 2 mg/kg for 2 months. Motor performance was assessed using rotarod testing and composite phenotype scoring, 3 and 9 weeks following the dosing regimen. **Results:** Preliminary results showed that a biweekly dosing at 2 mg/kg for 2 months improved the motor performance of the animals in rotarod and composite phenotype scoring tests in a short-term experiment. **Conclusions:** Despite being a small-scale study, our findings suggest that when treated early in life, the experimental GALT mRNA is effective in improving the motor-related phenotypes in *GalT*-KO mice using the specified dosing regimen. These findings highlight the potential of mRNA-based therapies for mitigating neurological symptoms in Classic galactosemia.

## 1. Introduction

**Figure 1:**
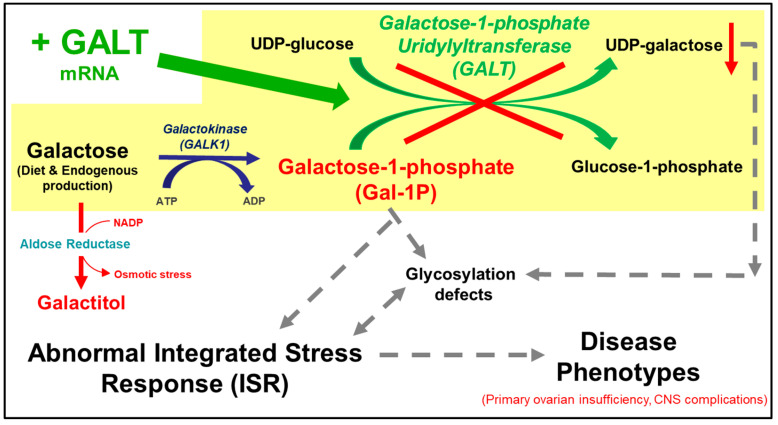
The Leloir pathway of galactose metabolism and the proposed pathobiology of galactose-1-phosphate uridylyltransferase (GALT) deficiency. Galactose, absorbed via diet or produced endogenously in a cell, is phosphorylated to form galactose-1-phosphate (Gal-1P). In the absence of GALT, the build-up of Gal-1P is thought to induce abnormal integrated response (ISR), which plays a role in the pathophysiology of the disease. Administration of *GALT* mRNA (green arrow) is expected to restore expression of GALT enzyme, thus effectively eliminating the Gal-1P accumulation.

Classic galactosemia, as defined in the Online Mendelian Inheritance in Man (OMIM) (230400), is an autosomal recessive disorder caused by a deficiency in galactose-1-phosphate uridylyltransferase (GALT, EC 2.7.7.12) activity [1,2,3,4,5]. GALT is the second enzyme in the evolutionarily conserved Leloir pathway of galactose metabolism, which catalyzes the simultaneous conversion of uridine diphosphoglucose (UDP-glucose) and galactose-1-phosphate (Gal-1P) to uridine diphosphogalactose (UDP-galactose) and glucose-1-phosphate (Figure 1) [6]. GALT deficiency leads to accumulation of toxic galactose metabolites such as Gal-1P and deficiency of UDP-galactose in patient cells [7,8]. If undiagnosed and untreated, classic galactosemia can be lethal for the affected newborns [3]. Since the inclusion of this disease in the newborn screening panel in the U.S., neonatal mortality has decreased [9]. The mainstay of treatment is the withdrawal of galactose from the diet [1]. However, despite early and adequate dietary management, endogenous production of galactose and its toxic metabolites continues [10,11] and patients have long-term complications such as intellectual deficits in 6-year-olds or older (45% of all patients), speech delay in 3-year-olds or older (56% of all patients), motor functions deficits (tremors and cerebellar ataxia) in 5-year-olds or older (18% of all patients), and primary ovarian insufficiency (POI) (91% of all female patients) [12,13]. Significantly reduced bone mineralization is increasingly seen in pre-pubertal patients of both sexes [14,15,16]. Except for the ovarian phenotype, there is considerable variability among other long-term complications. To-date, there is no effective treatment available to prevent or alleviate any of the above-mentioned long-term complications. Yet, regardless of the pathophysiological mechanisms, no one will argue that the root cause of the disease is the absence of GALT enzyme activity in patient cells. Therefore, therapeutic strategies that aim to restore GALT enzyme activity in the patients represent a rational and direct approach to address the unmet medical needs of the patients. Among them, experimental *GALT* mRNA therapy has emerged as a promising modality [17,18,19]. Indeed, we demonstrated significant efficacy of *GALT* mRNA in normalizing the disease-relevant biomarkers and restoring whole-body galactose oxidation in a mouse model of classic galactosemia.

In this short-term study, we expanded our preclinical proof-of-concept studies to include motor impairment, a disease-relevant phenotype. By doing so, we aimed to address the following three questions:

1. Will experimental *GALT* mRNA therapy improve motor-related phenotypes in a mouse model of classic galactosemia?
2. Will the improvement, if any, of motor-related phenotypes seen in the treated animals be sustained after the cessation of the experimental *GALT* mRNA treatment?
3. Does it matter if the *GALT* mRNA is offered to a younger versus an older mouse?

## 2. Materials and Methods

### 2.1. mRNA and Sterile Lipid Nanoparticle Synthesis and Formulation

mRNA was synthesized and formulated in sterile lipid nanoparticles (LNPs) as described previously [18]. The same biodegradable, ionizable LNPs described in our previous studies was used in the current set of studies [18]. Briefly, sequence-optimized mRNA encoding *GALT* was synthesized in vitro using optimized T7 RNA polymerase-mediated transcription reaction with complete replacement of uridine by N1-methyl-pseudoridine, following Moderna’s proprietary therapeutic protocol. The linearized DNA template contains the 5′ and 3′ untranslated regions (UTRs), open reading frame (ORF), and the poly-A tail. Inverted deoxythymidine (idT) residues were appended to the 3′ terminus of fully synthesized mRNA by phosphodiester linkage of a modifying oligo with 3′ inverted deoxythymidine (3′idT) [18,20]. The mRNA was produced with a 5′ cap 1 structure (cap1) to improve translation efficiency. Free and end-stabilized mRNA were purified, buffer exchanged, and sterile filtered. The mRNAs were tested for purity and capping efficacy and were found to be >70% and >90% effective, respectively. All formulations were tested for particle size, RNA encapsulation, and endotoxin presence and were found to be <100 nm in size, with >80% encapsulation, and <10 EU/mL endotoxin.

### 2.2. Animal Model

All animal protocols and procedures were approved and conducted in full compliance with the guidelines outlined in the Guide for the Care and Use of Laboratory Animals and were approved by the University of Utah Institutional Animal Care and Use Committee (IACUC). GalT-deficient (*GalT*-KO) mice used in this study were constructed as previously described [21] and were not challenged with galactose since weaning. Mice were housed in standard laboratory cages within temperature (22–23 °C) and humidity (30–70%) controlled rooms under a 12:12 light–dark cycle. All mice were confirmed by molecular genotyping using previously published protocol [21]. Both male and female animals were used for the present study and were uniformly distributed among each experiment group. All WT and *GalT*-KO animals were weaned at 3 weeks of age, at which point they were enrolled in the experiment.

### 2.3. In Vivo GALT mRNA Administration

**Figure 2:**
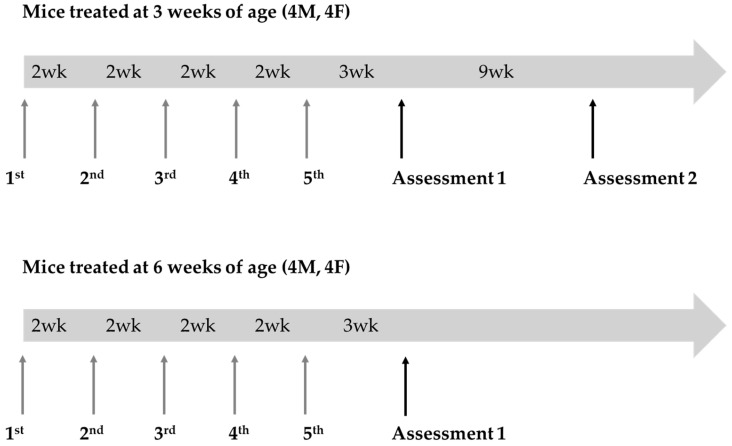
Experimental design for GALT mRNA treatment in GalT-KO mice. Two cohorts of GalT-KO mice (*n* = 8 per cohort; 4 males, 4 females) received five biweekly doses (2 mg/kg) of GALT mRNA. Treatment began at 3 weeks of age for cohort 1 and 6 weeks of age for cohort 2. Rotarod and composite phenotype scoring tests were conducted 3 and 9 weeks after the final mRNA dose for cohort 1 and 3 weeks for cohort 2 to assess motor function. Cohort 1 animals were 14 weeks old at assessment 1 and 23 weeks old at assessment 2. Animals in cohort 2 were 17 weeks old when assessed. Age-matched WT control animals were tested alongside both cohorts during their assessments.

*GalT*-KO mice aged 3 and 6 weeks old were administered 100 µL of LNPs containing *GALT* mRNA via intravenous injection. The LNPs encapsulated mRNA were supplied by Moderna, Inc. (Cambridge, MA, USA). Each experimental group consisted of 8 mice (4 males and 4 females), which were randomly enrolled into treatment groups at time of weaning (Figure 2). The mice received five biweekly doses of *GALT* mRNA at a concentration of 2 mg/kg. No placebo or vehicle doses were administered to the wild type (WT) control or untreated *GalT*-KO groups for this study.

### 2.4. Rotarod Test

Mice underwent rotarod testing 3 and 9 weeks following the dosing regimen. The 6-lane rotarod and accompanying software (Conductor Science SA102) used in this study was purchased from Maze Engineers (Skokie, IL, USA). Mice first completed a training session on the rotarod where they would walk at a constant speed of 4 rotations per minute (rpm) for at least 180 s before starting the first trial. Three trials were conducted where the rotarod would increase from 4 to 40 rpm at a constant acceleration of 7.2 rotations per minute squared (rpm^2^). Maximum speed would be reached at 300 s, which was also the cutoff time for each trial. Mice would remain on the stationary rotarod between trials, where they were allowed a 5 min rest period before beginning the next trial. The trial would automatically end when IR floor sensors detected the animal to have fallen off the rod, at which point the latency would be recorded. Test investigators would manually end the trial when a mouse was observed to complete two passive rotations, classified by the mouse holding onto the rod as it makes a full rotation without walking. To combat small sample sizes, the latency from all three trials were used to compare mRNA-treated *GalT*-KO results to WT control and untreated *GalT*-KO performances. The WT control median latency was used to categorize “under-performing” and “over-performing” animals within each treatment group. Chi-squared analysis was then utilized to compare the performance distribution, or the number of animals in each group to fall into these two categories. Statistical significance was achieved when the number of over- and under-performing animals in either *GalT*-KO group did not follow the expected distribution demonstrated by WT control animals. Results from chi-squared tests were verified using generalized linear models (GLMs) and post hoc assessments run in R 4.4.2 software.

### 2.5. Composite Phenotype Scoring Test

**Figure 3:**
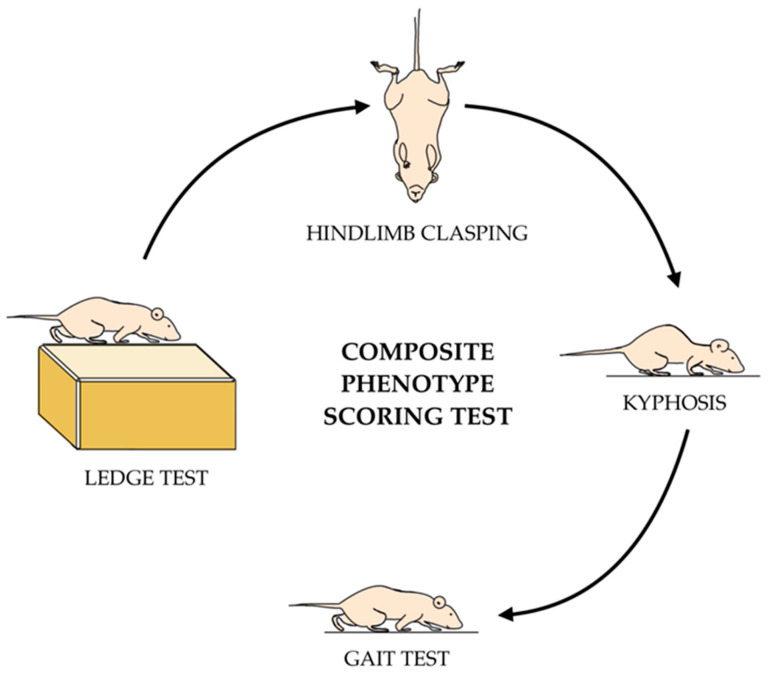
Schematic of the composite phenotype scoring test. Animals are scored on a 1–3 point scale for each test. Scores from the ledge, hindlimb clasping, kyphosis, and gait tests are summed together to obtain a total composite score for each mouse. A higher score indicates more of the disease phenotype.

The composite phenotype scoring test was conducted in accordance with the protocol demonstrated by Guyenet et al. (Figure 3) [22]. This assessment consists of four parts—the ledge test, hindlimb clasping, kyphosis, and gait tests—each measured on a 0–3 scale. The overall composite score is the combined scores from each of the four assessments, where a higher score signifies a more severe manifestation of disease phenotype (score 0–12). Two blinded investigators were assigned to evaluate the composite score test where they would average the two scores given if they differed. Half-scores were granted when the behavior did not align with the full-digit score.

The ledge test requires only the home cage of the mouse and is a simple assessment of motor coordination. Each mouse was gently placed on the ledge of the cage so that its hindlimbs can grasp onto the surface. If the mouse has no obvious issues with balance and can fully support its body while walking along the ledge, it receives a score of 0. If the mouse is observed to have several missteps as it walks on the ledge but is otherwise able to maintain its balance, it receives a score of 1. A score of 2 is granted if the hindlimbs are unable to support the body and instead the mouse is dragging along the ledge using only its front limbs. Alternately, if the mouse can support its body with several missteps but demonstrates a hard landing, it is scored as 2. A hard landing is defined by the animal falling onto its head instead of its front paws when lowering itself back into the home cage. If it is incapable of walking along the ledge despite all encouragement or continues to fall off without taking a full step, this will be scored a 3.

The hindlimb clasping test is conducted by lifting the mouse from the base of its tail approximately 5–10 cm off the ground and observing the position of the hind legs. If the hindlimbs remain splayed outward from the abdomen, the mouse will be given a score of 0. A score of 1 is classified by the mouse retracting one of its legs in towards the abdomen for more than 50% of suspension time. If both hindlimbs are partially retracted towards the abdomen, it will be given a score of 2. Lastly, if both legs are completely retracted toward the abdomen, this will result in a score of 3.

The gait test serves to evaluate motor function and coordination. Mice are placed on the flat surface of a biosafety cabinet (NUAIRE Biological Safety Cabinets, Plymouth, MN, USA) and observed by investigators. If an animal fully supports itself and evenly distributes its weight while walking, it will receive a score of 0. If there is a slight limp or abnormal stride noted, a score of 1 is granted. If the mouse is seen to have its pelvis tilted inward while walking, or demonstrates “duck feet”, it obtains a score of 2. The most severe score is granted when the pelvis is severely tilted inward, causing the animal to drag along the surface while walking, or a combination of less severe abnormalities (duck feet, limp, or uneven weight distribution).

Kyphosis is classified as the loss of spinal muscle tone and is a common symptom of neurodegeneration [22]. Mice are observed in both a sitting and walking position to assess kyphosis. If mild or no kyphosis is observed while sitting and the spine can fully straighten as the mouse walks, it will receive a score of 0. If mild kyphosis is exhibited while sitting but the mouse is still able to straighten its spine while walking, it will be scored a 1. If the mouse maintains mild kyphosis as it walks it will be given a 2, and if severe kyphosis is observed while sitting and walking, it scores a 3. R software was utilized to assess any changes in composite scoring behavior between treatment groups.

### 2.6. Statistical Analysis

Data are expressed as means ± standard error of the mean (SEM). Microsoft Excel, GraphPad Prism 9 (GraphPad Software, Inc., Boston, MA, USA) and R 4.4.2 software were used to analyze data. Chi-squared analysis was utilized to determine the differences in latency distribution in rotarod performances and a Mann–Whitney U test was used for composite phenotype scoring analysis between groups. R software was used to corroborate significant findings from these analyses.

## 3. Results

### 3.1. Near-Term Improvement in Motor Functions of GALT mRNA-Treated GalT-KO Mice

To address if experimental *GALT* mRNA therapy improves motor-related phenotypes in a mouse model of classic galactosemia (question 1), we compared the rotarod and composite phenotype scoring results from assessment 1 between the WT, treated, and untreated *GalT*-KO animals in cohort 1 (*n* = 8; 4 males and 4 females). Upon rotarod testing, untreated WT control mice demonstrated a median latency of 106.2 s (Table 1a). WT control median latency was used to define the performances of other groups, where any treated or untreated *GalT*-KO mouse with a latency less than the WT median was labeled as “under-performing”. Similarly, animals that demonstrated greater latencies than the WT median values were classified as “over-performing”.

**Figure 4:**
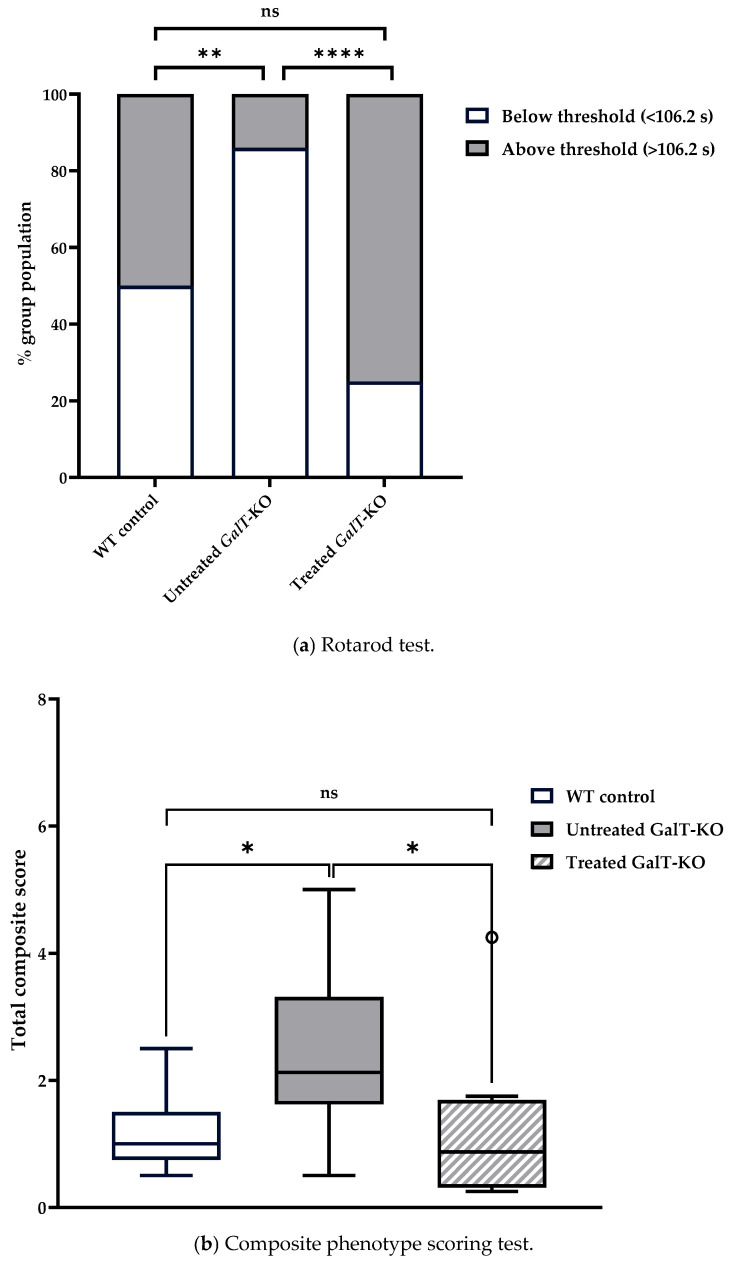
Motor function evaluation of cohort 1 at 3 weeks after the last dose of *GALT* mRNA treatment. (**a**) Rotarod performance distribution 3 weeks after the end of treatment. The graph displays the percentage of animals in each treatment group that performed either below or above the WT control median latency (threshold). Chi-squared testing was used to determine whether the performance distribution of treated or untreated GalT-KO animals differed from WT control mice. (**b**) Composite phenotype scoring 3 weeks after treatment ended. The box plot shows the total composite score of each treatment group, reflecting overall motor function and behavior. The central line denotes the median, and the whiskers represent the range of scores. Data points beyond the whiskers represent outliers in each treatment group which fall outside of the 25–75th percentile. All data points, including outliers, were included in statistical analyses. Data are presented as means ± SEM (ns = not significant; * *p* < 0.05; ** *p* < 0.01; **** *p* < 0.0001). Statistical analyses were performed using Mann–Whitney U Test. Influence of treatment group, gender, and other variables on rotarod and composite score performances were analyzed by running GLMs with Gaussian distribution in R programming (see Supplementary Materials).

We then compared the number of animals that were categorized as “under-performing” and “over-performing” across treatment groups using chi-squared analysis. In assessment 1, 85.7% of untreated *GalT*-KO mice performed under the WT median latency, compared to only 25% of mRNA-treated *GalT*-KO animals (Figure 4a). When we examined the distribution of untreated and mRNA-treated *GalT*-KO animals based on the WT control median values, we saw a significant improvement in the treated animals (*p* = 0.00005). Untreated mutant mice showed a drastically different distribution compared to the WT control animals (*p* = 0.001). Treatment of the *GalT*-KO animals proved to be effective in diminishing the genotypic difference. A GLM with a Gaussian distribution was run using treated *GalT*-KO mice as the reference group to confirm results obtained by chi-squared testing. This model confirmed our previous findings by showing that untreated *GalT*-KO animals are estimated to perform 49.37 s worse than the treated *GalT*-KO group (*p* = 0.0002) (Figure S1). Furthermore, post hoc pairwise comparison further validated these results with the untreated *GalT*-KO animals having a significantly lower latency than both WT control and treated *GalT*-KO groups (*p* = 0.0005 and *p* = 0.013, respectively) (Table S1a,b). Body weight and sex were not found to be influential factors on latency in this assessment.

We continued to see significant distinctions between the performances of the untreated WT and *GalT*-KO during the composite phenotype scoring test. Untreated WT control mice had a median combined score of 1, and untreated *GalT*-KO mice scored significantly higher (more disease phenotype) with a median score of 2.13 (*p* = 0.046) (Table 1b). mRNA-treated *GalT*-KO mice showed improvement and scored substantially lower than the untreated *GalT*-KO cohort with a median score of 0.88 (*p* = 0.05) (Figure 4b). Besides showing better performance than the untreated mutant group, the mRNA treatment of *GalT*-KO mice also demonstrated a return to the expected performance of WT animals (*p* = 0.71). To validate these findings, R software was used to run a model assessing the effect of treatment and gender on composite score performance. This model confirmed that untreated *GalT*-KO animals performed significantly worse than the treated *GalT*-KO group (*p* = 0.003), and that sex of the untreated *GalT*-KO animals was an influential factor on outcome (*p* = 0.0344) (Table S1c). Post hoc analysis confirmed the significant improvements in mRNA-treated *GalT*-KO female animals compared to their untreated counterparts (*p* = 0.0344) (Table S1d). Considering the rotarod and composite phenotype scoring test results, we concede that mRNA treatment of 3-week-old *GalT*-KO mice does improve their motor function.

### 3.2. Improvement in Motor Functions from a Single Dose of GALT mRNA Therapy Were Not Sustained Far Beyond Three Weeks

**Figure 5:**
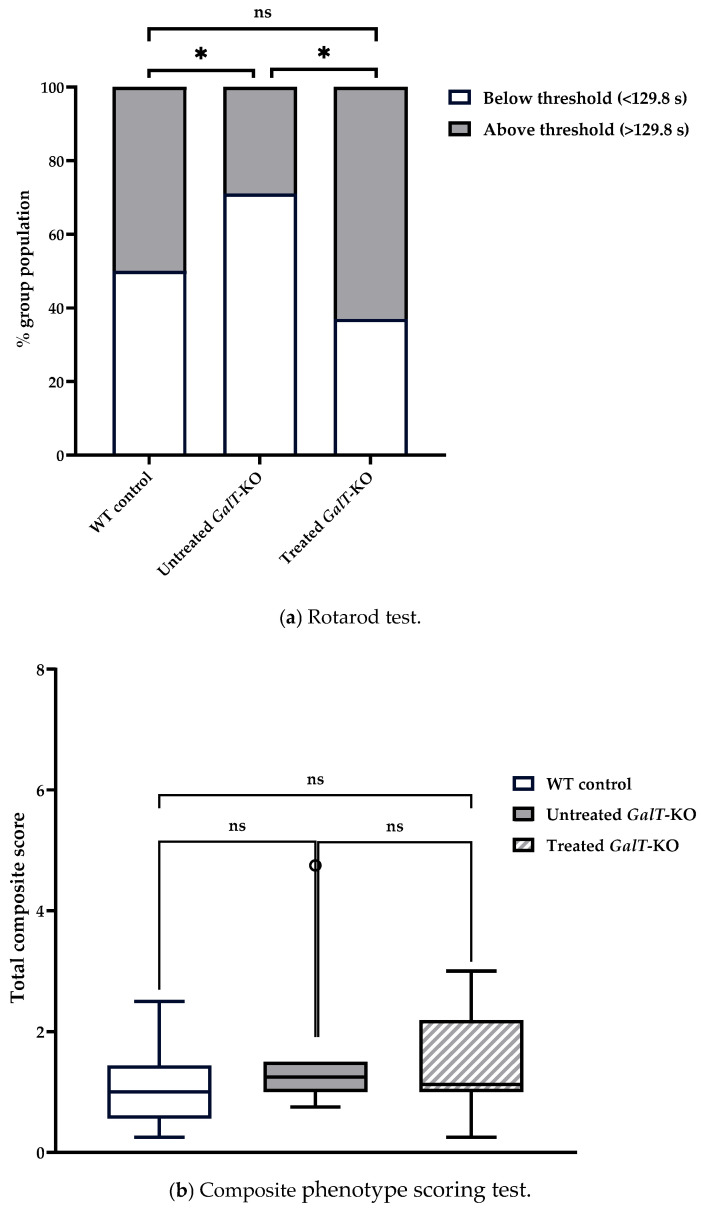
Motor function evaluation of cohort 1 at 12 weeks after GALT mRNA treatment ended. (**a**) Rotarod performance distribution of WT treated and untreated animals. The majority of treated GalT-KO animals performed above the WT control group median latency, whereas the untreated mutant animals mostly performed under this threshold. (**b**) Composite phenotype scoring test yielded no significantly different performances between treatment groups. WT and untreated GalT-KO groups demonstrated an improved mean composite score from the first assessment, while mRNA-treated animals maintained their performance. Data represent mean ± SEM. Statistical significance was determined using Mann–Whitney U test. General linearized models with Gaussian distribution were run in R to determine the effect of treatment type on rotarod and composite scoring test performances (see Supplementary Materials). ns = not significant, * *p* < 0.05.

To see if the positive changes resulting from *GALT* mRNA treatment are sustainable (question 2), we re-tested these mice after 9 weeks, or when they reached 23 weeks of age (Figure 2). For rotarod testing, untreated WT control mice had a median latency of 130 s, while untreated *GalT*-KO mice had a median latency of 95.5 s (Table 1a). We saw 71.4% of the untreated mutant mice performing under the WT median threshold, demonstrating a significant genotypic difference in performance distribution (*p* = 0.049) (Figure 5a). Treated *GalT*-KO mice had a median latency of 137.5 s and showed significant difference in their distribution when compared to the untreated *GalT*-KO cohort (*p* = 0.023) (Figure S2). While the untreated mutant mice mostly performed under the WT median latency, we found that only 37.5% of the mRNA-treated *GalT*-KO group fell below this threshold (Figure 5a). No difference was noted between WT- and mRNA-treated mutant group distribution (Figure 5a). A GLM with Gaussian distribution confirmed treatment type to have significant influence on latency. Untreated *GalT*-KO animals were estimated to perform 14.44 s worse than WT controls and 32.41 s worse than treated *GalT*-KO animals (Figure S2). This model also substantiated results from chi-squared analysis, showing that treated *GalT*-KO group still demonstrated significantly higher latencies than their untreated counterparts, 12 weeks following the end of their treatment (*p* = 0.011) (Table S2a). Again, post hoc testing in R confirmed this distinction. Though a significantly better performance was still observed in the treated *GalT*-KO group at this timepoint, the magnitude of effect is notably smaller than obtained in assessment 1. Comparing the two assessments, there was no apparent decline in the mRNA-treated *GalT*-KO group performance. Instead, we saw a trend of increased latency in the untreated mutant group over time (Figures S1 and S2). With these results, we infer that the benefits from mRNA treatment of *GalT*-KO animals may still exist at this point, though the impact is surely decreasing.

In the composite score phenotype scoring test, untreated WT mice demonstrated a score near to their first assessment, with a median total score of 0.875 (Table 1b). Untreated and mRNA-treated *GalT*-KO groups performed similarly with median composite scores of 1.25 and 1.125, respectively (Table 1b). Compared to assessment 1, both WT and untreated *GalT*-KO groups improved their scores, while the treated mutant group showed a slight decline in performance (Figure 5b). There were no distinctions between any of the three groups in this assessment, as confirmed with the Mann–Whitney U test and models run in R (Table S2c). Consequently, we cannot confidently confirm the benefits of mRNA treatment in *GalT*-KO animals have sustained over time for this test.

### 3.3. GALT mRNA Treatment Appears to Be More Effective in Early Life

**Figure 6:**
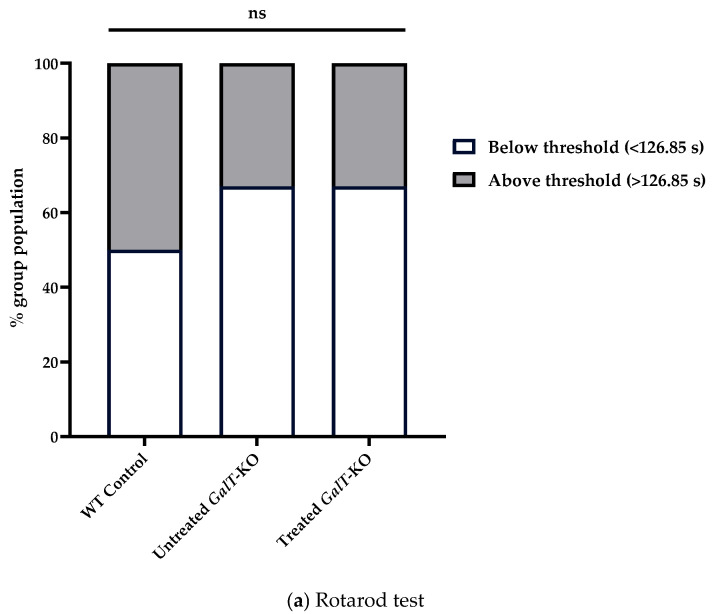

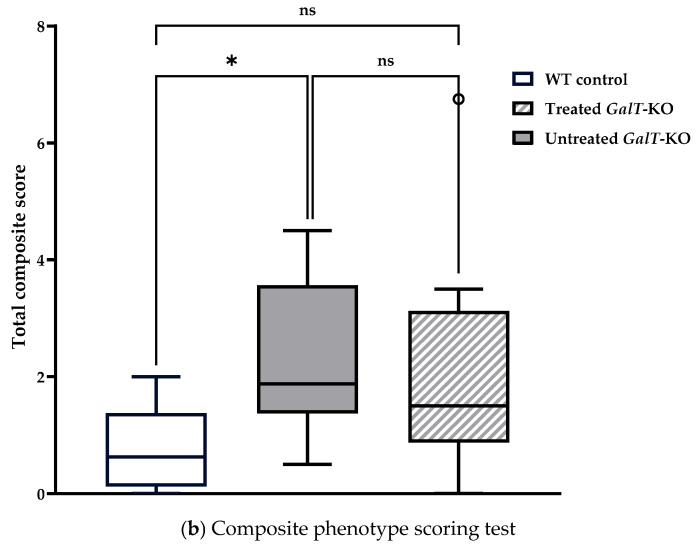
Motor function assessment in aged mice following GALT mRNA treatment administered at 6 weeks of age. (**a**) Rotarod test results demonstrated no difference between treated and untreated *GalT*-KO performance relative to WT median latency. Chi-squared analysis was utilized to determine significance in rotarod performance distribution. (**b**) Composite phenotype score evaluation indicated there was no comparable performance between untreated *GalT*-KO mice and mRNA-treated *GalT*-KO animals. Data represent mean ± SEM. Statistical significance was determined using Mann–Whitney U test. ns = not significant; * *p* < 0.05.

Finally, we compared the above results with separate groups of mice that we began dosing at an older age to address question 3 (6 weeks of age) (Figure 2). For rotarod testing, untreated WT control mice had a median latency of 126.9 s, while untreated *GalT*-KO mice had a median latency of 101.9 s (Table 1a). Despite the mRNA-treated *GalT*-KO group showing a slightly higher median latency (122.6 s) than their untreated counterparts (Figure S3), both treated and untreated *GalT*-KO groups followed the same performance distribution relative to the WT threshold latency (Table 1a, Figure 6a). We could not differentiate these groups as 66.7% of each population performed under the WT median latency (Figure 6a). A GLM confirmed the lack of influence that mRNA treatment had on latency in this assessment (Table S3a). While the treatment of 6-week-old *GalT*-KO animals did not show any improvements in rotarod performance, we observed that the untreated *GalT*-KO animals performed much better in the 6-week versus 3-week cohort.

For the composite phenotype scoring test, untreated WT control mice had a median total score of 0.625, and untreated *GalT*-KO mice had a significantly higher median score of 1.875 (*p* = 0.021) (Table 1b). mRNA-treated *GalT*-KO mice performed similarly, with a median total score of 1.5 (Table 1b). Unlike the untreated mutant group, however, the treated *GalT*-KO animals showed no significant deviation from WT median scores (Figure 6b). Neither treatment group nor gender elicited a significant influence on composite scoring abilities, as confirmed by GLMs run in R (Table S3).

## 4. Discussion

Recent advances and successes in molecular therapeutics have paved the way for revolutionary treatment modalities for many monogenic recessive diseases such as phenylketonuria and classic galactosemia [19,23,24,25,26,27]. Among these modalities, mRNA-based therapies are gaining ground because the successful track record in vaccine development. Additionally, such treatment, if successful, will address the root cause of the diseases, thus making them rational choices when one aims for a potentially curative therapy. In fact, at least two mRNA-based programs have made their way to clinical trials after promising preclinical studies. NCT04899310 is a study to assess safety, pharmacokinetics, and pharmacodynamics of mRNA-3705 in participants with isolated methylmalonic acidemia. This was accomplished after years of in vivo POC work [28,29,30]. Similarly, NCT05095727 is a study of mRNA-3745 in adult and pediatric participants with glycogen storage disease type 1a (GSD1a). This was also achieved after substantial in vivo POC work [31].

In this study, we aimed to extend our earlier in vivo POC work for an experimental *GALT* mRNA therapy for classic galactosemia from disease-relevant [17] and functional biomarker [18] normalization to disease-relevant phenotypes correction in a mouse model of galactosemia. As we have previously shown that these *GalT*-KO animals manifest motor-related impairment [32], we focused on the potential improvement of this phenotype.

As a pilot investigation, we chose to test one dosing regimen; this consisted of a biweekly dosing of 2 mg mRNA-LNP formulation per kg of body weight for two months before we commenced the behavioral assessments (rotarod and composite phenotype scoring tests). We chose biweekly administration because it will affect the compliance of patients if they have to receive such treatment more than once every two weeks. We selected 2 mg/kg because it was determined in our earlier studies that such a dosage is safe and a single IV dose of this specific *GALT* mRNA is effective in maintaining a normal level of galactose metabolites in liver of the mutant mice up to 2 weeks [18].

We opted for a two-month-long treatment because we wanted a short-term multi-dose study. Enrolled animals were dosed starting at 21 and 42 days old via tail-vein injections, repeating biweekly for two months. Behavioral assessments were conducted at 3 and 9 weeks following the final mRNA dose to assess motor impairment phenotypes.

Baseline behavioral assessments were not carried out for this study as cohort 1 began their dosing immediately after weaning, and many potentially confounding physiological changes will occur after the dosing is completed. Instead, results were directly compared between age- and sex-matched treatment groups. Moreover, previous experiments conducted by our lab suggest that motor impairment phenotypes as assessed by rotarod and composite phenotype scoring tests are not detectable until our mouse model reaches at least breeding age (6–8 weeks). This was another reason for us proceeding without pre-treatment assessments.

Based on the results from assessment 1 for mice treated at 3 weeks of age, we clearly saw some significant improvements in rotarod and composite phenotype scoring tests in the first assessment of the younger mutant mice treated with the *GALT* mRNA, when they were 14 weeks of age (Figure 4). Paradoxically, we saw an improvement in the performances of the untreated mutant mice for both rotarod test (i.e., increased latency) and composite phenotype scoring test (i.e., lower combined score) overtime (i.e., in assessment 2) when the animals reached 23 weeks of age (close to 6 months) (Figure 5). Although such improvement has wiped out much of the genotypic differences between WT and untreated *GalT*-KO mice that we saw in the first assessment (Figure 4), mRNA-treated animals continued to perform above the WT median latency and significantly outperformed the untreated *GalT*-KO animals (*p* = 0.023) (Figure 5a). Comparing the rotarod in assessments 1 and 2, we do not detect any change in performance in the mRNA-treated animals, indicating that the treatment effects have likely sustained.

Although there is no notable decline in performance in the treated group, we do see that the gap between untreated and treated *GalT*-KO animals is much smaller. In assessment 1, we observed extremely high significance when testing the difference between treated and untreated *GalT*-KO groups (*p* = 0.0002), and untreated *GalT*-KO animals were estimated to perform 49.37 s worse than treated animals (Table S1a). In assessment 2, we still saw a substantially worse performance in the untreated *GalT*-KO animals compared to treated *GalT*-KO, though to a much smaller degree with the untreated group estimated to score 32.41 s lower (*p* = 0.011) (Table S2a). mRNA treatment of *GalT*-KO animals seems to sustain its impact on the rotarod test and allows treated mutant mice to perform at WT levels nine weeks after treatment was finished. Though the improvement is still visible, we see a declining trend where the difference between treated and untreated animals is becoming less significant over time.

Importantly, results from Figure 6 revealed that for the dosing regimen tested, it appears no significant improvements were achieved when we dosed the animals at 6 weeks of age. However, it could be due to the assessment of the animals at an older age, which somehow showed a trend towards improvement regardless of treatment. Nevertheless, our data did suggest that it will be more effective to commence the mRNA treatment at a younger age (i.e., 3-week-old) (Figure 4). No significant influence of treatment was detected when running linear regression models on these results (Table S3b).

Despite being a small-scale study, our data indicated that when treated early in life, the experimental *GALT* mRNA is effective in improving the motor-related phenotypes in *GalT*-KO mice using the specified dosing regimen. Moreover, our data suggests that due to the finite half-life of mRNA, repeated dosing should be considered to sustain the long-term positive changes. At first glance, repeated dosing might represent a disadvantage over other modalities like Adeno-associated Virus (AAV)-mediated gene transfer [33,34], which are usually aimed for once in a lifetime administration. However, mRNA therapy also offers an attractive and practical alternative to patients who have pre-existing immunity against AAV, short-term therapeutic needs, and less widespread disease phenotypes.

While the improved outcomes with treatment initiation at 3 weeks may suggest a therapeutic window, we agree that this could also reflect baseline phenotype severity as we compared the results with the untreated mutant mice, and such windows may be different for different phenotypes. Finally, it would be interesting to extend the current study to other disease-relevant phenotypes like subfertility in the future.

## 5. Limitations

Despite promising results, our studies presented here are not without limitations. First, small group sizes reduce statistical power and may limit the detection of subtle treatment effects. Second, for some investigators, the absence of placebo LNP controls may be less ideal, although untreated animals or human subjects have been routinely used as controls. Third, the lack of a pre-treatment baseline assessment for individual mice limits our ability to measure within subject changes over time, which could have strengthened the interpretation of therapeutic effects. However, the lack of phenotypes in young mice motivated us to skip such assessment in this study. Lastly, we concede that we have not performed any correlation testing between the various biochemical biomarkers and GALT activity or protein abundance in these animals. Yet, we have conducted extensive pharmacokinetic and pharmacodynamic analyses of our specific *GALT* mRNA species in our previous work [17,18], and therefore, we focused on the phenotypic analyses in this study. We will address all these concerns in future studies when similar investigations are conducted.

## Figures and Tables

**Table 1:** Descriptive statistics tables created in R programming depicting (**a**) rotarod and (**b**) composite phenotype scoring assessments. The “vars” column represents the number of variables (i.e., treatment type, cohort); “SD” is the standard deviation of the dataset; “MAD” is the median absolute deviation; “SE” is standard error of the mean; and “Trimmed” represents the trimmed mean, or the mean after removing the 10% highest and lowest data points (outliers). MAD is the median absolute deviation, measuring the average distance from the median.

(a) Descriptive statistics from rotarod assessments.															
Treatment Group	Treatment Age	Assessment No.	vars	*n*	Mean	SD	Median	Trimmed	MAD	Min	Max	Range	Skew	Kurtosis	SE
WT Control	3 weeks	1	1	24	109.0	45.7	106.2	107	28.5	16.1	221	205	0.477	0.334	9.33
Untreated *GalT*-KO	3 weeks	1	1	21	72.8	37.3	64.0	68.4	25.9	20.2	164	144	1.080	0.317	8.14
Treated *GalT*-KO	3 weeks	1	1	24	122.0	40.8	134.7	124	30.2	43.9	178	134	−0.661	−0.748	8.33
WT Control	6 weeks	1	1	24	118.0	53.5	126.9	115	73.9	46.7	244	198	0.465	−0.813	10.9
Untreated *GalT*-KO	6 weeks	1	1	24	104.0	35.3	101.2	103	42.5	46.4	174	127	0.202	−0.987	7.2
Treated *GalT*-KO	6 weeks	1	1	24	116.0	30.6	122.6	117	20.8	46.7	162	115	−0.602	−0.408	6.26
WT Control	3 weeks	2	1	24	118.0	40.1	130.0	119	41.3	48.4	179	131	−0.202	−1.240	8.19
Untreated *GalT*-KO	3 weeks	2	1	21	104.0	40.4	95.5	100	37.8	52.5	196	143	0.687	−0.734	8.81
Treated *GalT*-KO	3 weeks	2	1	24	136.0	44.3	137.5	135	34.5	49	268	219	0.617	1.420	9.05
(**b**) **Descriptive statistics from composite phenotype scoring assessments.**															
**Treatment Group**	**Treatment Age**	**Assessment No.**	**vars**	***n***	**Mean**	**SD**	**Median**	**Trimmed**	**MAD**	**Min**	**Max**	**Range**	**Skew**	**Kurtosis**	**SE**
WT Control	3 weeks	1	1	8	1.19	0.651	1.00	1.19	0.556	0.5	2.5	2	0.79	−0.693	0.23
Untreated *GalT*-KO	3 weeks	1	1	8	2.44	1.35	2.13	2.44	0.927	0.5	5	4.5	0.492	−0.797	0.479
Treated *GalT*-KO	3 weeks	1	1	8	1.28	1.32	0.88	1.28	0.927	0.25	4.25	4	1.28	0.322	0.466
WT Control	6 weeks	1	1	8	0.78	0.7	0.63	0.781	0.741	0	2	2	0.435	−1.32	0.247
Untreated *GalT*-KO	6 weeks	1	1	8	2.25	1.35	1.88	2.25	0.741	0.5	4.5	4	0.515	−1.29	0.477
Treated *GalT*-KO	6 weeks	1	1	8	2.16	2.11	1.50	2.16	0.927	0	6.75	6.75	1.13	−0.0184	0.747
WT Control	3 weeks	2	1	8	1.09	0.706	1.00	1.09	0.556	0.25	2.5	2.25	0.697	−0.691	0.25
Untreated *GalT*-KO	3 weeks	2	1	7	1.71	1.36	1.25	1.71	0.371	0.75	4.75	4	1.49	0.546	0.516
Treated *GalT*-KO	3 weeks	2	1	8	1.47	0.881	1.13	1.47	0.741	0.25	3	2.75	0.382	−1.27	0.311

## Data Availability

Data supporting the studies presented in this manuscript can be made available upon request to the corresponding authors.

## Supplementary Materials

The following supporting information can be downloaded at https://www.mdpi.com/article/10.3390/biomedicines13122848/s1. Figure S1. Rotarod latency distribution of 3-week treatment groups. Boxplot showing the latency distribution for cohort 1 during their first rotarod assessment. *GalT*-KO animals treated at 3 weeks with mRNA displayed the highest latency out of the three groups, followed by WT controls. Untreated *GalT*-KO animals performed the worst in assessment 1. The midline in each box represents the median latency and all datapoints outside of the box parameters are defined as outliers, though they are still included in analyses. The box boundaries represent points within the 25th to 75th percentile of our data. Significance levels were determined by running one-way ANOVA and post-hoc pairwise comparisons between groups. Table S1. Results from cohort 1 first assessment at 14 weeks of age. (a) Generalized linear model (GLM) with Gaussian distribution assessing the effect of treatment type on latency, using mRNA-treated *GalT*-KO animals as the reference group. The coefficients column represents the group in comparison with the reference. ‘Intercept’ is the baseline predicted mean of the reference group, used to predict the difference in mean outcomes between groups (estimate). The T value is the ratio of the estimate to the standard error of the mean and is meant to be compared to the T distribution curve to determine statistical significance based on degrees of freedom. (b) Post-hoc analysis on 3-week rotarod GLM findings executed in R 4.4.2 software. ‘Estimate’ column values are the estimated difference in latency (s) between the groups in comparison. ‘df’ refers to the degrees of freedom, or the number of observations minus the parameters in the model and is used alongside the T value to determine statistical significance. (c) Results from a GLM using box-cox transformed composite score data from cohort 1 at 3 weeks after treatment. mRNA-treatment and sex were shown to be significant factors influencing the performance of *GalT*-KO animals. (d) Post-hoc analysis on assessment 1 composite phenotype scoring test from cohort 1. Improvement in this test was found to be prevalent in female GalT-KO mice only. Figure S2. Rotarod latency distribution by treatment groups of cohorts 1 in assessment 2. Boxplot showing the median (center line), interquartile range (IQR) (box edges), values ranging within 1.5X the IQR (whiskers), and outlier (individual dots) latency recordings for treatment groups in cohort 1 assessed at 23 weeks old. Treated *GalT*-KO mice demonstrated significantly longer latency times compared to untreated mutant animals 9 weeks after dosing ended. Table S2. (a) GLM with Gaussian distribution assessing rotarod performances of cohort 1 in assessment 2. The ‘Estimate’ column shows the estimated difference of latencies between the reference (treated GalT-KO) group to the compared group. The T value is the ratio of the estimate to the standard error and is used to determine the P value based on the T distribution curve and degrees of freedom. (b) Post-hoc analysis on rotarod assessment 2 GLM. ‘SE’ is standard error, and ‘df’ is the degrees of freedom in the corresponding comparison. (c) GLM assessing composite phenotype scoring test results and treatment interactions of cohort 1 in assessment 2. No significant influence of treatment type on composite score performance was observed. Figure S3. Rotarod performances of cohort 2 animals assessed at 17 weeks old. Box and whiskers graph (Tukey style) displaying rotarod performances of cohort 2 animals. No change in latency was observed between any treatment group, confirmed with one-way ANOVA testing. Table S3. (a) Results from a GLM with Gaussian distribution analyzing rotarod performances of cohort 2. (b) GLM results for the composite phenotype scoring assessment of 6-week treated animals.
