# Supplementary material for: Experimental Galactose-1-Phosphate Uridylyltransferase (GALT) mRNA Therapy Improves Motor-Related Phenotypes in a Mouse Model of Classic Galactosemia—A Pilot Study
Source: Biomedicines. 2025 Nov 21;13(12):2848. doi: 10.3390/biomedicines13122848 (PMC12731057; doi:10.3390/biomedicines13122848)
Supplement: Supplementary file 1 [file biomedicines-13-02848-s001.zip › biomedicines-3817722-supplementary.pdf]

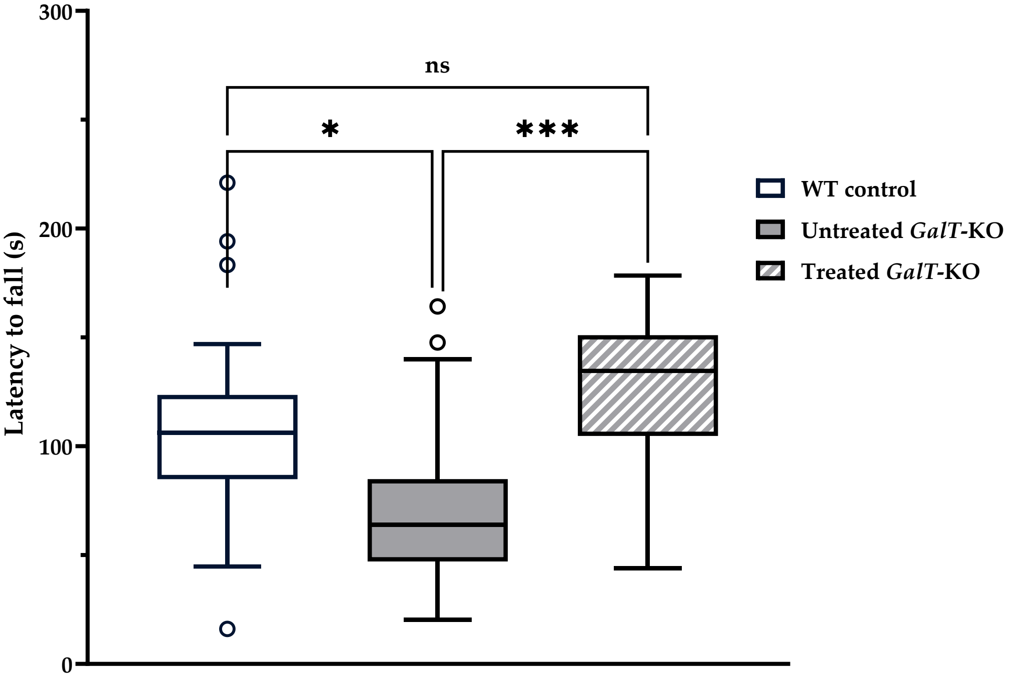

Figure S1. Rotarod latency distribution of 3-week treatment groups. Boxplot showing the latency distribution for cohort 1 during their first rotarod assessment. *GalT*-KO animals treated at 3 weeks with mRNA displayed the highest latency out of the three groups, followed by WT controls. Untreated *GalT*-KO animals performed the worst in assessment 1. The midline in each box represents the median latency and all datapoints outside of the box parameters are defined as outliers, though they are still included in analyses. The box boundaries represent points within the 25th to 75th percentile of our data. Significance levels were determined by running one-way ANOVA and post-hoc pairwise comparisons between groups.

Table S1. Results from cohort 1 first assessment at 14 weeks of age.

(a) Generalized linear model (GLM) with Gaussian distribution assessing the effect of treatment type on latency, using mRNA-treated *GalT*-KO animals as the reference group. The coefficients column represents the group in comparison with the reference. 'Intercept' is the baseline predicted mean of the reference group, used to predict the difference in mean outcomes between groups (estimate). The T value is the ratio of the estimate to the standard error of the mean and is meant to be compared to the T distribution curve to determine statistical significance based on degrees of freedom.

Coefficients	Estimate	SE	T value	P value
Intercept	122.179	8.492	14.388	< 2E-16
Untreated *GalT*-KO	-49.370	12.430	-3.972	0.000179
WT control	-13.062	12.009	-1.088	0.280667

(b) Post-hoc analysis on 3-week rotarod GLM findings executed in R 4.4.2 software. 'Estimate' column values are the estimated difference in latency (s) between the groups in comparison. 'df' refers to

the degrees of freedom, or the number of observations minus the parameters in the model and is used alongside the T value to determine statistical significance.

Comparison	Estimate	SE	df	T value	P value
Treated *GalT*-KO – Untreated *GalT*-KO	49.4	12.4	66	3.972	0.0005
Treated *GalT*-KO – WT control	13.1	12.0	66	1.088	0.5250
Untreated *GalT*-KO – WT control	-36.3	12.4	66	-2.921	0.0131

(c) Results from a GLM using box-cox transformed composite score data from cohort 1 at 3 weeks after treatment. mRNA-treatment and sex were shown to be significant factors influencing the performance of *GalT*-KO animals.

Coefficients	Estimate	SE	T value	P value
Intercept	-0.6678	0.3538	-1.887	0.07533
Untreated *GalT*-KO	1.6837	0.5003	3.365	0.00345
WT control	0.6183	0.5003	1.236	0.23242
Sex: Male	1.1522	0.5003	2.303	0.03343
Untreated *GalT*-KO: Male	-1.5867	0.7076	-2.242	0.03776
WT control: Male	-0.8991	0.7076	-1.271	0.22000

(d) Post-hoc analysis on assessment 1 composite phenotype scoring test from cohort 1. Improvement in this test was found to be prevalent in female *GalT*-KO mice only.

Comparison	Estimate	SE	df	T value	P value
Treated *GalT*-KO females – Untreated *GalT*-KO females	-1.684	0.5	18	-3.365	0.0344
Treated *GalT*-KO females – WT control females	-0.618	0.5	18	-1.236	0.8140
Untreated *GalT*-KO females – WT control females	1.065	0.5	18	2.129	0.3165
Treated *GalT*-KO males – Untreated *GalT*-KO males	-0.097	0.5	18	-0.194	1.000
Treated *GalT*-KO males – WT control males	0.281	0.5	18	0.561	0.9924
Untreated *GalT*-KO males – WT control males	0.378	0.5	18	0.755	0.9717

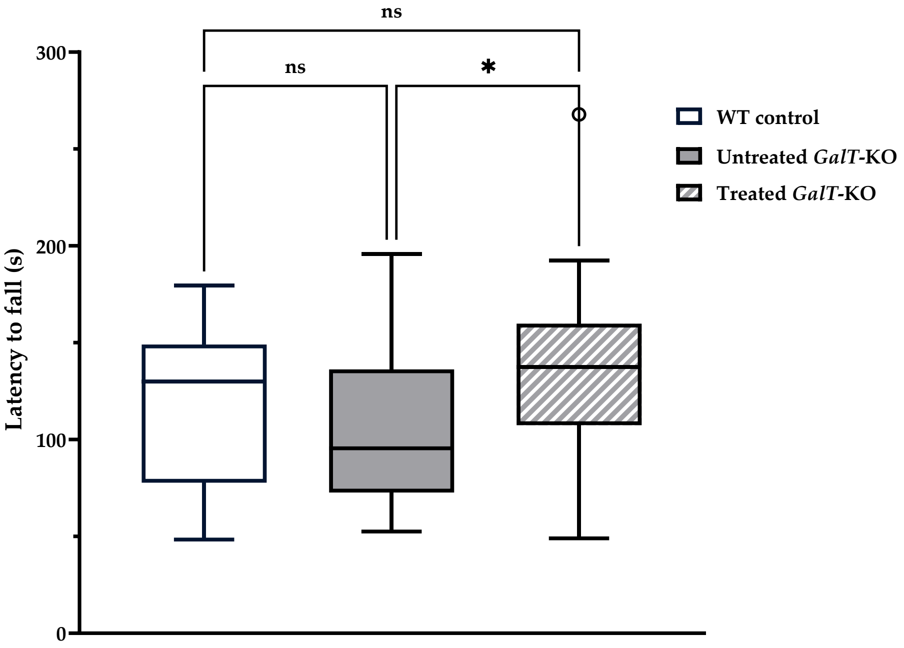

Figure S2. Rotarod latency distribution by treatment groups of cohort 1 in assessment 2. Boxplot showing the median (center line), interquartile range (IQR) (box edges), values ranging within 1.5X the IQR (whiskers), and outlier (individual dots) latency recordings for treatment groups in cohort 1 assessed at 23 weeks old. Treated *GalT*-KO mice demonstrated significantly longer latency times compared to untreated mutant animals 9 weeks after dosing ended.

Table S2.

(a) GLM with Gaussian distribution assessing rotarod performances of cohort 1 in assessment 2. The 'Estimate' column shows the estimated difference of latencies between the reference (treated GalT-KO) group to the compared group. The T value is the ratio of the estimate to the standard error and is used to determine the P value based on the T distribution curve and degrees of freedom.

Coefficients	Estimate	SE	T value	P value
Intercept	132.638	8.512	26.529	< 2E-16
Untreated *GalT*-KO	-32.438	12.461	-2.612	0.0111
WT control	-17.973	12.038	-1.453	0.1509

(b) Post-hoc analysis on rotarod assessment 2 GLM. 'SE' is standard error, and 'df' is the degrees of freedom in the corresponding comparison.

Comparison	Estimate	SE	df	T value	P value
Treated *GalT*-KO – Untreated *GalT*-KO	32.4	12.5	66	2.612	0.0295
Treated *GalT*-KO – WT control	17.9	12.0	66	1.453	0.3200
Untreated *GalT*-KO – WT control	-14.5	12.5	66	-1.209	0.4524

(c) GLM assessing composite phenotype scoring test results and treatment interactions of cohort 1 in assessment 2. No significant influence of treatment type on composite score performance was observed.

Coefficients	Estimate	SE	T value	P value
Intercept	0.2393	0.2463	0.972	0.343
Untreated *GalT*-KO	0.1713	0.3606	0.475	0.640
WT control	-0.2971	0.3484	-0.853	0.404

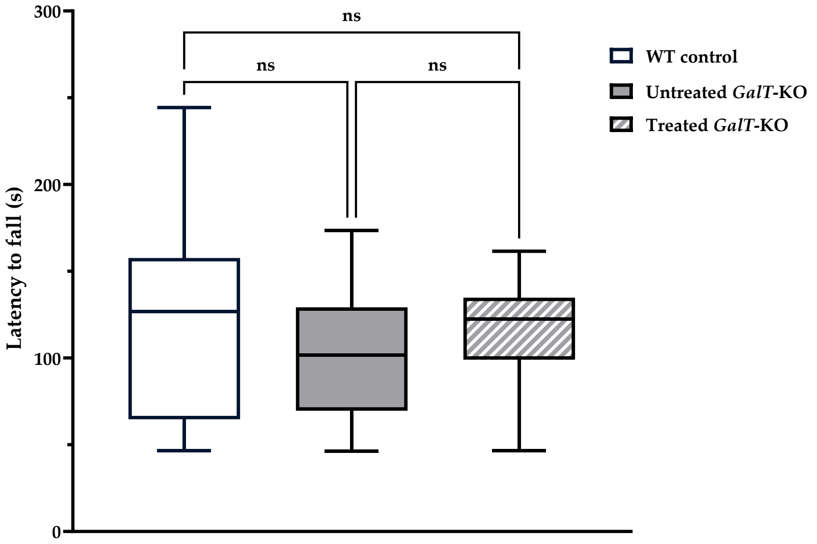

Figure S3. Rotarod performances of cohort 2 animals assessed at 17 weeks old. Box and whiskers graph (Tukey style) displaying rotarod performances of cohort 2 animals. No change in latency was observed between any treatment group, confirmed with one-way ANOVA testing.

Table S3.

(a) Results from a GLM with Gaussian distribution analyzing rotarod performances of cohort 2.

Coefficients	Estimate	SE	T value	P value
Intercept	115.583	8.367	13.814	< 2E-16
Untreated *GalT*-KO	-11.492	11.833	-0.971	0.335
WT control	2.854	11.833	0.241	0.810

(b) GLM results for the composite phenotype scoring assessment of 6-week treated animals.

Coefficients	Estimate	SE	T value	P value
Intercept	0.5347	0.4025	1.328	0.1983
Untreated *GalT*-KO	0.3056	0.5693	0.537	0.5971
WT control	-1.0602	0.5693	-1.862	0.0766
